# Structural Characterization and Evaluation of Interfacial Properties of Pea Protein Isolate–EGCG Molecular Complexes

**DOI:** 10.3390/foods11182895

**Published:** 2022-09-18

**Authors:** Shuang Han, Fengzhan Cui, David Julian McClements, Xingfeng Xu, Cuicui Ma, Yutang Wang, Xuebo Liu, Fuguo Liu

**Affiliations:** 1 College of Food Science and Engineering, Northwest A&F University, Xianyang 712100, China; 2 Department of Food Science, University of Massachusetts Amherst, Amherst, MA 01003, USA; 3 College of Food Science and Engineering, Qingdao Agricultural University, Qingdao 266109, China

**Keywords:** pea protein isolate, EGCG, interaction, interfacial properties

## Abstract

**Highlights:**

- Pea protein isolate (PPI) and EGCG spontaneously formed complexes.
- Protein–polyphenol complexation was mainly driven by hydrogen bonding.
- The binding of EGCG influenced the structure and functionality of PPI.
- PPI-EGCG complexes had better emulsifier properties than PPI.

**Abstract:**

There is increasing interest in using plant-derived proteins in foods and beverages for environmental, health, and ethical reasons. However, the inherent physicochemical properties and functional performance of many plant proteins limit their widespread application. Here, we prepared pea protein isolate (PPI) dispersions using a combined pH-shift/heat treatment method, and then, prepared PPI-epigallocatechin-3-gallate (EGCG) complexes under neutral conditions. Spectroscopy, calorimetry, molecular docking, and light scattering analysis demonstrated that the molecular complexes formed spontaneously. This was primarily ascribed to hydrogen bonds and van der Waals forces. The complexation of EGCG caused changes in the secondary structure of PPI, including the reduction in the α-helix and increase in the β-sheet and disordered regions. These changes slightly decreased the thermal stability of the protein. With the accretion of EGCG, the hydrophilicity of the complexes increased significantly, which improved the functional attributes of the protein. Optimization of the PPI-to-EGCG ratio led to the complexes having better foaming and emulsifying properties than the protein alone. This study could broaden the utilization of pea proteins as functional ingredients in foods. Moreover, protein–polyphenol complexes can be used as multifunctional ingredients, such as antioxidants or nutraceutical emulsifiers.

## 1. Introduction

Concerns about the adverse effects of animal-derived foods on the environment, health, and religion are leading to increasing interest in plant-based diets [1,2]. Consequently, many researchers are examining the potential of replacing animal-derived functional ingredients with plant-derived alternatives, especially plant-based proteins [3,4]. Pea protein has high nutritional value, low price, and low allergenicity, and is therefore an important source of plant-based proteins in food formulations [5]. Pea protein isolate (PPI) has a total protein content exceeding 90% with a nutritionally balanced amino acid profile and a range of health benefits, including anti-inflammatory, antioxidant, and cholesterol-lowering properties [6,7,8]. However, the high proportion of insoluble globulin molecules in pea protein (about 70%) results in its poor solubility, which, in turn, causes poor functions, such as emulsification, foaming, and gelling [9,10]. For this reason, researchers are aiming to broaden the utilization of pea protein in foods and beverages by using physicochemical methods, such as sonication, homogenization, pH-shift, enzymatic catalysis, and complexation methods [11,12].

Polyphenols are secondary metabolites of edible plants, and can reduce the oxidative damage that occurs in proteins, lipids, and carbohydrates in active cells and tissues [13]. After ingestion, they may also exhibit beneficial health effects within the human body, thereby acting as nutraceuticals. Tea polyphenols (TPs) are well-known sources of natural antioxidants, of which epigallocatechin-3-gallate (EGCG) has the strongest biological activity. It has been demonstrated that EGCG has excellent antioxidant, anti-inflammatory, and anticancer activities in preclinical studies, but its application is often hindered due to its sensitivity to light, heat, and pH in foods, and simultaneously, its low bioavailability in the gastrointestinal tract [14,15].

In food matrices, polyphenols can combine with proteins to form soluble complexes that enhance their stability [7]. Moreover, the binding of polyphenols to proteins can improve their physicochemical properties and functional performance, such as emulsifying, foaming, gelling, and digestibility properties [16,17]. For example, Jia et al. [18] reported that EGCG increased the foaming and emulsifying properties of whey proteins, and Jiang et al. [19] reported that chlorogenic acid improved the solubility and digestibility of caseins and whey proteins. Many protein–polyphenol complexes are dual-function ingredients that can be used as both emulsifiers and antioxidants. These complexes are able to adhere to oil–water interfaces and restrain oxidation reactions in emulsified foods, and can also act as both emulsifiers and nutraceuticals in functional food and beverage products [20,21].

The interactions between proteins and polyphenols are divided into covalent and non-covalent types. Conjugates are formed by forming strong covalent bonds using chemical or enzymatic methods. In contrast, complexes are formed through physical interactions, for example, hydrogen bonding, van der Waals and/or electrostatic forces [22]. Covalent bonds are stronger than non-covalent ones, but undesirable by-products may be generated during their formation, such as quinone polymers. Moreover, the creation of new ingredients using covalent bonding often requires regulatory approval, which is expensive and time-consuming. Therefore, there is great interest in expanding protein–polyphenol complexes as functional components in the food industry [5,23].

This study focused on the utilization of physical interactions to form soluble PPI-EGCG molecular complexes that exhibit functional performance superior to PPI alone. Information on PPI-EGCG interactions was obtained using spectroscopy, calorimetry, and molecular docking analysis. In addition, the influence of complexation on the physicochemical characteristics and functional performance of the pea proteins were determined. Our work could increase the application of pea proteins in foods. Moreover, it provides valuable information on the nature of protein–polyphenol non-covalent interactions, which may aid in the reasonable design of protein–polyphenol complexes in the future.

## 2. Materials and Methods

### 2.1. Materials

Pea protein isolate (PPI, purity ≥ 90 wt%) was obtained from Zelong Biotechnology Co., Ltd. (Xi’an, China). Epigallocatechin-3-gallate (EGCG, purity ≥ 95 wt%) was obtained from BSZH Scientific Inc. (Beijing, China). Corn oil was obtained from COFCO Fulinmen Food Marketing Co., Ltd. (Shanghai, China). 8-Anilino-1-naphthalenesulfonic acid (ANS) was purchased from Aladdin Holdings Group Co., Ltd. (Beijing, China). Aqueous solutions were prepared using purified distilled water throughout the study.

### 2.2. Preparation of PPI Dispersions and PPI-EGCG Complexes

The PPI dispersions were prepared according to Zhang et al. (2012) [24] with slight modifications. Briefly, 1.0 g of PPI was added to 100 mL of distilled water and stirred for 30 min at 25 °C. The pH of this mixture was then adjusted to 12.0 using 2 M NaOH and left for 30 min. This alkaline-treated PPI sample was then heated at 85 °C for 30 min to allow the structure of the pea protein molecules to unfold. The resulting solutions were then cooled to 25 °C and transferred to pH 7.0 with 2 M HCl to obtain the PPI dispersions.

Different concentrations of EGCG aqueous solutions were freshly prepared using phosphate buffer saline (PBS, 10 mM, pH 7.0) solution. Subsequently, PPI-EGCG complexes were prepared by mixing the PPI dispersion with equal volumes of EGCG solution and stirring for 2 h in the dark at 25 °C. The PPI solutions and PPI-EGCG complexes were made into powder via freeze-drying (LC-10N-50A, lichen, Shanghai, China).

### 2.3. Spectroscopic Characterization of PPI-EGCG Complexes

#### 2.3.1. Fluorescence Spectroscopy

The fluorescence spectra of the aqueous dispersions of the PPI and PPI-EGCG complexes were measured using a fluorescence spectrophotometer (F-7000, Hitachi, Chiyoda, Japan). The final concentrations of PPI and EGCG were 1.5 mg/mL and 0 to 50 μM, respectively. The excitation wavelengths were 280 and 295 nm, respectively. The emission spectrum range was 300–500 nm, and both the excitation and emission band widths were 5 nm. Variable-temperature fluorescence spectra were obtained at 298, 304, and 310 K. Synchronous fluorescence spectra were obtained at Δλ = 15 and 60 nm at 25 °C. The spectrum of an aqueous buffer solution containing the same amount of EGCG was removed from the sample spectrum for baseline correction.

#### 2.3.2. UV Spectroscopy

The UV spectra of the samples were obtained using a UV spectrophotometer (UV-2550, Shimadzu, Kyoto, Japan) in the wavelength range of 250–350 nm. The final concentrations of PPI and EGCG were 1.5 mg/mL and 0–100 μM, respectively. A phosphate buffer was used as a blank control.

#### 2.3.3. Circular Dichroism (CD)

The CD spectra of the aqueous dispersions of the samples were acquired using a CD spectropolarimeter (Chirascan V100, Applied Photophysics, Britain) in the far-UV (190–250 nm) region. The PPI and EGCG concentrations used were 0.2 mg/mL and 0–100 µM, respectively. The instrument operating conditions were set at a 0.2 nm step resolution, 1 nm bandwidth and 60 nm/min speed. The CD spectra of PBS and aqueous buffer solutions with different EGCG concentrations were removed from the corresponding sample spectra for baseline correction. The percentage content of the secondary structures was calculated using the instrument software CDpro.

#### 2.3.4. Fourier Transform Infrared Spectroscopy (FTIR)

A Fourier transform infrared spectrophotometer (Vetex70, Bruker, Bremen, Germany) was used to measure the infrared spectroscopy of the PPI and PPI-EGCG complexes. Briefly, 1 mg powdered sample and 100 mg of KBr were mixed in an agate mortar and pressed to obtain transparent sample discs. The FTIR spectra of the samples were measured at wavelengths of 400 to 4000 cm^−1^ and scanned 32 times at a resolution of 4 cm^−1^.

### 2.4. Thermodynamic Characterization of PPI-EGCG Complexes

#### 2.4.1. Isothermal Titration Calorimetry (ITC)

The thermodynamic properties of the PPI-EGCG complexes were investigated according to Zhan et al. [25] using an ITC microcalorimeter (Nano ITC, Waters, New Castle, DE, USA) at 25 °C. All samples were vacuum degassed for 10 min before each titration. The PPI (1 mg/mL) was added to the reaction cell (300 µL) and EGCG (6 mM, 8 mM, or 10 mM) was added to the syringe (50 µL). After equilibrium, a series of 25 consecutive 0.2 µL EGCG solutions were injected for titration. The first injection was not used in the results because of leakage problems from the syringe. There was a 180 s delay between the continuous injections, and 300 rpm agitation was maintained in the experiment to ensure adequate mixing. The calibration experiments included titrating the EGCG solutions into PBS (no PPI). The concentration of PPI was indicated as mg/mL because PPI is a blend of proteins. So, the enthalpy change (kJ) per mol of injectant (ΔH, kJ/mol) versus the ratio of EGCG (mmol) to PPI (g) was plotted to show the results.

#### 2.4.2. Differential Scanning Calorimetry (DSC)

The thermal stability of the PPI and PPI-EGCG complexes was obtained using a DSC instrument (Q2000, Waters, New Castle, DE, USA). Freeze-dried samples (3 mg), which were prepared from solutions containing 1.5 mg/mL PPI and 0–100 μM EGCG, were sealed in aluminum pans for measurement. An empty, sealed aluminum pan was used as a blank. A temperature scan was then carried out from 40 to 200 °C at 10 °C/min. The maximum transition temperature (Tmax) was determined using the instrument software.

### 2.5. Molecular Docking of PPI and EGCG

The protein and polyphenol were docked using AutoDock Tools to further understand their interaction mechanism. The target protein structure was based on the reported 11S pea protein (ID: 3KSC), the main component of PPI, which was downloaded from the Protein Data Bank (PDB). The structure of EGCG (CAS: 989-51-5) was retrieved from the Pubchem database. PyMOL software (PyMOL 2.5, Schrödinger, New York, NY, USA) was used to remove non-amino acid residues in the proteins, and AutoDock was used for processing. Polar hydrogen and a Gasteiger charge were added to the protein molecules prior to analysis. After processing, the docking area was selected and the semi-flexible docking program Set Rigid Filename (receptor protein is rigid, and the ligand polyphenol is flexible) was used for calculation [26]. The best-scoring pose with the least energy was selected and analyzed using PyMoL software.

### 2.6. Physicochemical Properties of PPI-EGCG Complexes

#### 2.6.1. Surface Hydrophobicity (H_0_)

An extrinsic fluorescent probe (1-anilinonaphthalene- 8-sulfonic acid, ANS) was used to obtain the surface hydrophobicity of the samples [27]. Briefly, the protein concentrations in PPI dispersions and PPI-EGCG complexes with different EGCG concentrations were diluted to 0.05–0.25 mg/mL. Then, 20 μL ANS (8 mM) was added to the 4 mL sample for fluorescence determination at 390 nm, an emission range of 400–600 nm, and slit widths of 5 nm. H_0_ is the slope of the image of fluorescence intensity versus PPI concentration (mg/mL).

#### 2.6.2. Turbidity

The turbidity of aqueous dispersions was measured utilizing a UV spectrophotometer (UV-1240, Shimadzu, Kyoto, Japan) at 600 nm. Different concentrations of EGCG solutions were combined with the PPI solution to reach final mass ratios of PPI-to-EGCG of 4:1, 6:1, 8:1, 10:1, 12:1, and 14:1 with a fixed PPI content of 1 mg/mL. The PBS was used as a blank.

#### 2.6.3. Particle Properties

The size, polydispersity index (PDI), and zeta-potential of particles in the PPI and PPI-EGCG complex dispersions with different PPI-to-EGCG mass ratios were measured using a Zetasizer (ZEN3600, Malvern, Worcestershire, UK).

### 2.7. Interfacial Properties of PPI-EGCG Complexes

#### 2.7.1. Foaming Properties

The foaming properties of the PPI and PPI-EGCG complexes were measured using the method stated by Sui et al. [28]. Briefly, 15 mL of each sample was sheared (10,000 rpm, 1 min) using a high-shear homogenizer (Ultra-Turrax IKA-T25, Staufen, Germany) to generate foam. The foam volume was recorded immediately after shearing was stopped to calculate the foam expansion (FE). The foam stability (FS) was then measured after standing for 30 min. The FE and FS values were obtained according to the formulae below:

(1) $$\mathrm{FE}\left(\%\right)=\frac{V_{T}}{V_{O}}\times 100$$

(2) $$\mathrm{FS}\left(\%\right)=\frac{V_{t}}{V_{O}}\times 100$$

Here, V_T_ is the volume of the PPI and PPI-EGCG complex solution (mL) immediately after agitation, V_O_ is the volume of the solution (20 mL) before agitation, and V_t_ is the volume of the sample (mL) after standing for 30 min.

#### 2.7.2. Emulsifying Properties

The emulsifying properties of the PPI and PPI-EGCG complexes were determined based on the approach described by Meng & Li [29] with slight modifications. A total of 5 mL corn oil was added to the 15 mL sample and the mixture was homogenized (20,000 rpm, 1 min) using the high-shear homogenizer (Ultra-Turrax IKA-T25, Staufen, Germany). At 0 and 10 min, 100 μL of the samples were drawn from the bottom of the emulsion and mixed into 10 mL of 0.1% (*w*/*v*) sodium dodecyl sulfate (SDS) solution for dilution and floc disruption. Then, the absorbance of the mixture was obtained at 500 nm (0.1% *w*/*v* SDS solution as a blank). The emulsion activity index (EAI) and emulsion stability index (ESI) were then calculated using the formulae below:

(3) $$\mathrm{EAI}\left(m^{2}/g\right)=\frac{2\times 2.303\times A_{0}}{\emptyset \times C\times 10^{4}}$$

(4) $$\mathrm{ESI}\left(\min\right)=\frac{A_{0}\times 10}{A_{0}-A_{10}}\times 100$$

Here, A_0_ and A_10_ are the absorbance of the dispersions at 0 and 10 min; φ is the oil volume fraction; and C is the protein concentration (g/mL).

### 2.8. Statistical Analysis

All experiments were carried out in triplicate or more and the results are provided as the mean ± standard deviation. All figures were drawn using graphical analysis software (Origin 2019, Originlab Inc, Northampton, MA, USA). IBM SPSS Statistics 23.0 (IBM Corporation, Armonk, NY, USA) was used for analysis of variance at a *p*-level of 0.05.

## 3. Results and Discussion

### 3.1. Spectroscopic Analysis of PPI-EGCG Complexes

#### 3.1.1. Variable-Temperature Fluorescence Spectroscopy

A change in fluorescence intensity can indicate the accessibility of quenchers to protein fluorescent groups, which is helpful in recognizing the binding mechanism of proteins and ligands. Tryptophan (Trp) residues can be excited at an excitation wavelength of 280 and 295 nm, while tyrosine (Tyr) residues can only be excited at 280 nm. Quenching occurs when the quencher is close enough to tryptophan or/and tyrosine residues, and thus, the participation of Trp and Try in the complexes can be estimated by comparing the fluorescence quenching characteristics of proteins excited at different excitation wavelengths [25].

**Figure 1:**
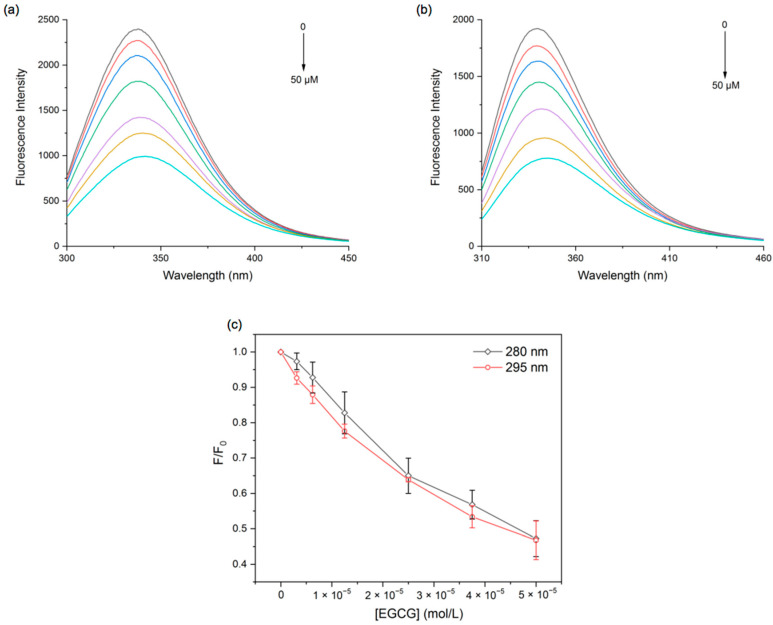
Fluorescence emission spectra of PPI at excitation wavelengths of 280 nm (**a**) and 295 nm (**b**), and fluorescence quenching spectra plots of PPI at 298 K (**c**) with different EGCG concentrations.

According to Figure 1a, the fluorescence intensity of PPI peaked at 338 nm at an excitation wavelength of 280 nm. As EGCG increased, the fluorescence emission intensity of PPI decreased regularly, and the maximum emission peak was red-shifted (338 nm–342 nm). Under the excitation wavelength of 295 nm, the spectrum of PPI showed a peak at 340 nm. Meanwhile, the peak red-shifted (340–345 nm) and the fluorescence intensity decreased with the increase in EGCG (Figure 1b), which had the same trend as the excitation wavelength of 280 nm. This suggests that the reaction of proteins with polyphenols resulted in protein molecular unfolding and amino acid residues exposed to a more hydrophilic environment [30]. Additionally, it can be seen from Figure 1c that the quenching spectra of the protein at 280 and 295 nm did not overlap, indicating that both Trp and Try were involved in the PPI and EGCG interactions. The quenching data were calculated using the Stern–Volmer equation:

(5) $$F_{0}/F=1+K_{\mathrm{sv}}\left[P\right]=1+K_{q}\tau_{0}\left[P\right]$$

Here, F_0_ is the fluorescence intensity in the absence of EGCG, while F is the fluorescence intensity in the presence of EGCG. K_SV_ is the Stern–Volmer quenching constant. [P] is the quencher concentration. K_q_ is the bimolecular quenching rate constant. τ_0_ is the lifetime (10^−8^ s) of fluorescence in the absence of the quencher.

The fluorescence quenching mechanisms involve static quenching (the formation of ground-state complexes), dynamic quenching (intermolecular diffusion and collision), and the coexistence of the two. It is generally accepted that static quenching is characterized by a K_q_ greater than 2.0 × 10^10^ L·mol^−1^s^−1^ and decreases in K_SV_ with increasing temperature, whereas the reverse is dynamic quenching [31]. In the present study, we found the K_q_ of PPI fluorescence quenching caused by EGCG was significantly larger than 2.0 × 10^10^ L·mol^−1^s^−1^, but the K_SV_ slightly increased when the temperature rose (Figure S1 and Table 1), indicating that the quenching mechanism was static quenching supplemented by dynamic quenching.

The binding constant (K_a_) and the number of binding sites (n) of EGCG molecules bound to PPI molecules were calculated using the equation below [32]:

(6) $$\mathrm{Log}\left[\frac{F_{0}-F}{F}\right]={\mathrm{LogK}}_{a}+\mathrm{nLog}\left[P\right]$$

The double logarithmic curves and the calculation results show that the binding sites of EGCG and PPI were all close to 1, suggesting that the two had at least one binding site. The binding constants of EGCG at different excitation wavelengths were all greater than 10^4^ L·mol^−1^, indicating that the binding forces between EGCG and PPI were stronger (Figure S2 and Table 2). It is beneficial to slow down the oxidation of polyphenols, prolong the life of polyphenols, and increase their bioavailability.

#### 3.1.2. Synchronous Fluorescence Spectroscopy

Protein fluorescence intensity is closely related to the polarity of its amino acid residues, and a change in the microenvironment of the aromatic amino acid residues leads to fluorescence quenching and a shift in the emission wavelength. In the synchronous fluorescence spectra of proteins, ∆λ = 15 nm and ∆λ = 60 nm represent the spectral properties of tyrosine and tryptophan residues, respectively [33].

**Figure 2:**
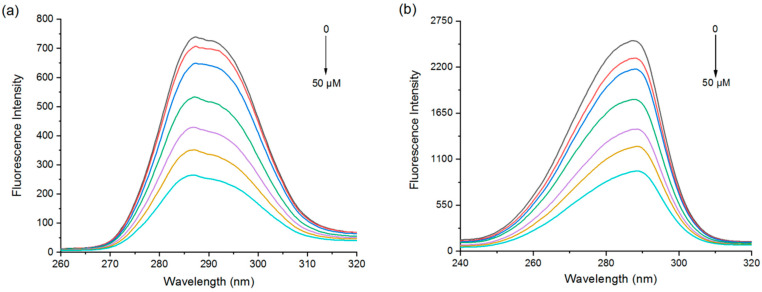
Synchronous fluorescence spectra of PPI at Δλ = 15 nm (**a**) and Δλ = 60 nm (**b**) with different EGCG concentrations.

The fluorescence intensity due to Try and Trp was gradually quenched with increasing EGCG concentration (Figure 2). The fluorescence wavelength peak of the tyrosine residues was slightly blue-shifted (288 to 287 nm) (Figure 2a), while that of the tryptophan residues was red-shifted (287 to 290 nm) (Figure 2b). Further analysis of the contribution of Tyr and Trp by deconvolution also found that Trp residues contributed more to the PPI and EGCG interactions (Figure S3). Combined with the fluorescence emission spectrum data, these results can illustrate that the polyphenol binding site was closer to the tryptophan residues than the tyrosine ones.

#### 3.1.3. UV Spectroscopy

**Figure 3:**
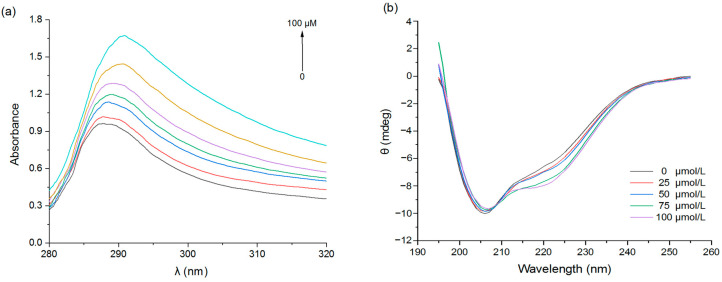
UV absorption spectra (**a**) and CD spectra (**b**) of PPI with different EGCG concentrations.

Changes in the intensity and position of UV absorption peaks can also reveal changes in the microenvironment of the hydrophobic amino acid residues in proteins [34]. With the increase in EGCG concentration, the intensity of the PPI absorption peak increased (Figure 3a), indicating increasing complexation between the EGCG and PPI molecules; moreover, the red-shift of the absorption peak was consistent with a change in the microenvironment of the hydrophobic protein groups after interacting with the polyphenols.

#### 3.1.4. Circular Dichroism (CD)

Secondary structure changes caused by the complexation of pea proteins with EGCG were determined using circular dichroism (CD). PPI had a distinct negative peak at 200–210 nm and a shoulder around 220 nm (Figure 3b). When EGCG was added, the ellipticity of the CD spectrum changed, indicating a change in the secondary structure of PPI. Further analysis revealed that the pure pea protein was composed of 27.0% α-helix, 16.2% β-sheet, 21.9% β-turn, and 35.0% random coil (Table 3). After binding with EGCG, the content of α-helix and random coil both decreased, the β-turn decreased slightly, and the β-sheet increased. These results suggest that some of the α-helix structure may have changed to β-sheet structure or may have become more disordered [35]. This effect may be because EGCG bound to the hydrophobic amino acid regions in the α-helix structure, thereby altering the spatial conformation of the pea protein molecules [36].

#### 3.1.5. Fourier Transform Infrared Spectroscopy (FTIR)

**Figure 4:**
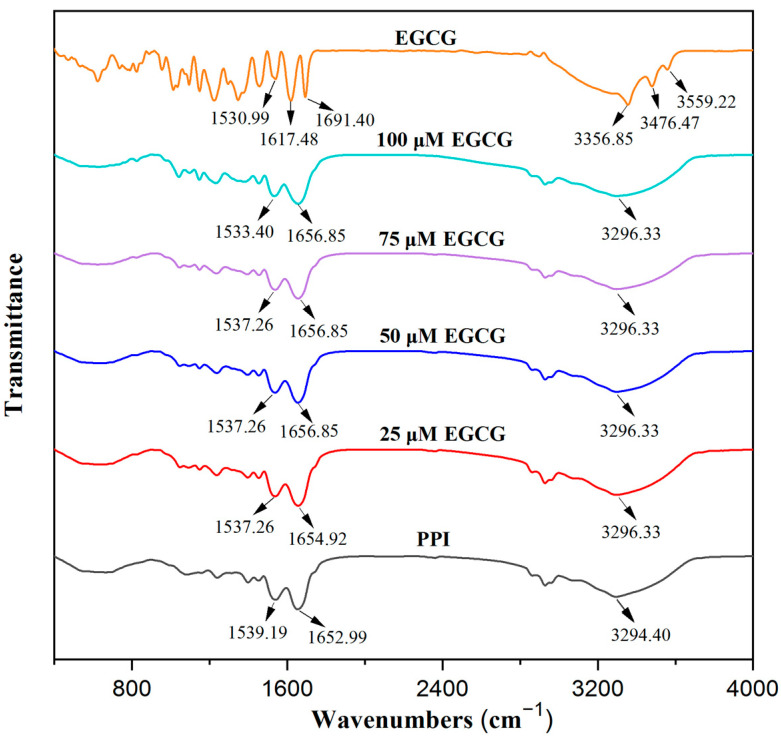
FTIR spectra of EGCG and PPI with different EGCG concentrations.

FTIR can be used to supply information about the structure changes and interactions of proteins by examining the intensity and position of characteristic spectral peaks [21]. EGCG had two peaks around 1691 and 1617 cm^−1^, probably due to the carbonyl stretching of gallic acid. The peaks around 3356, 3476, and 3559 cm^−1^ were attributed to O-H stretching of the phenolic hydroxyl groups [33]. The characteristic peaks of pure PPI were the amide I band (1600–1700 cm^−1^, C=O stretching vibration) around 1653 cm^−1^, the amide II band (1450–1550 cm^−1^, N-H bending and C-N stretching) around 1539 cm^−1^, and the amide A band (hydrogen bonding and N–H stretching vibrations) around 3294 cm^−1^ (Figure 4). After the interaction between EGCG and PPI, the amide A band of PPI was red-shifted (the O-H stretch of EGCG was blue-shifted) to 3296 cm^−1^, which was due to hydrogen bond formation between the protein and polyphenol molecules. The amide I band of PPI red-shifted from 1653 to 1655 cm^−1^ when a small amount of EGCG was added, and then, further red-shifted to 1657 cm^−1^ when a high polyphenol concentration was added. The amide II band blue-shifted from 1539 to 1537 cm^−1^ when a small amount of EGCG was added; then, it further blue-shifted to 1533 cm^−1^, which illustrates that the secondary structure of PPI changed [37]. These results show that the more EGCG was added, the greater the impact on PPI structure, which led to greater changes in the FTIR spectra.

### 3.2. Thermodynamics Analysis of PPI-EGCG Complexes

#### 3.2.1. Isothermal Titration Calorimetry (ITC)

**Figure 5:**
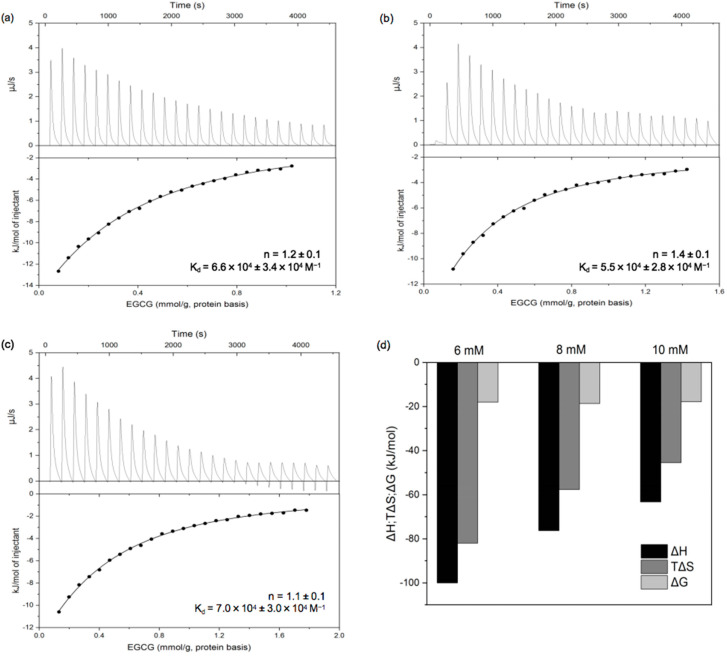
Thermograms (**top**) and binding isotherms (**bottom**) for titration of 6 mM (**a**), 8 mM (**b**), and 10 mM (**c**) EGCG into PPI dispersion (1 mg/mL) and thermodynamic profile of EGCG and PPI interaction (**d**).

Information about the thermodynamics of protein–polyphenol interactions can be obtained using isothermal titration calorimetry (ITC) using a single-point binding model based on the analysis of S-shaped curves. The heat flow versus time profiles and the corresponding binding isotherms of PPI solution injected with different concentrations of EGCG solution are shown in Figure 5a–c. We found that non-S-shaped curves were obtained, which is consistent with multiple interactions occurring between the proteins and polyphenols such as hydrophobic, hydrogen bonding, van der Waals forces, etc.; this, in turn, led to protein molecular aggregation, structure transition, and self-association/dissociation [29,38]. Together, these molecular interactions result in the emergence of non-S-shaped curves. The number of ECGC-to-protein binding sites (n) of was about 1, and the binding constant (K_d_) was more than 10^4^ M^−1^, which was similar to the results calculated from the fluorescence spectra. As shown in Figure 5d, the ΔH, ΔS, and ΔG values linked to the interactions between PPI and EGCG were all negative; this indicates that the binding process was spontaneous, and that the interaction was mainly driven by hydrogen bonds as well as van der Waals forces, presumably between -OH groups on the polyphenols and hydrogen bonding sites on the proteins. Additionally, ΔH > TΔS, indicating that the enthalpy change associated with the interaction was greater than the entropy change, which was mainly attributed to hydrogen bonding. The absolute values of ΔH and ΔS decreased with increasing EGCG concentration, but that of ΔG hardly changed. This suggests some enthalpy–entropy compensation associated with the binding interaction [36,39].

#### 3.2.2. Differential Scanning Calorimetry (DSC)

**Figure 6:**
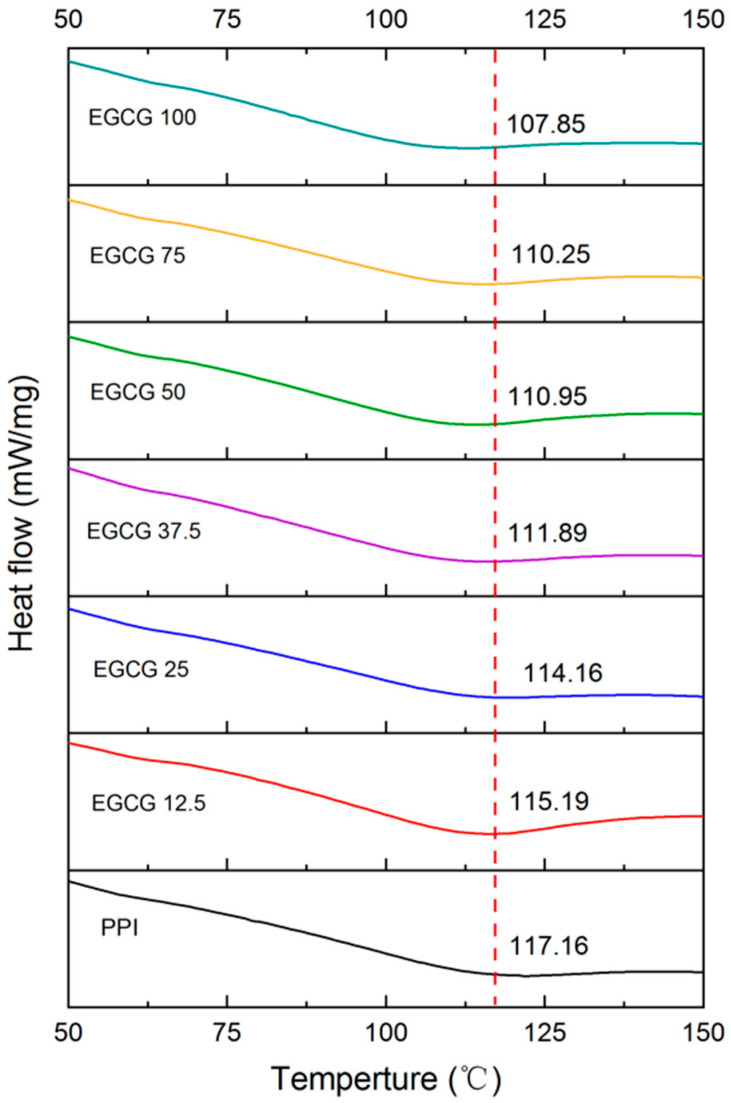
The heat flow curves of PPI with different EGCG concentrations.

Information about the impact of polyphenols on the thermal stability of proteins was obtained using DSC analysis, as the formation of complexes can significantly alter the thermal denaturation temperature (T_d_) of proteins. A change in a DSC curve can provide valuable information about a change in protein structure. The DSC profile of the pure PPI showed that it underwent a single endothermic transition with a peak temperature of around 117.2 °C. The presence of this peak indicates that the protein underwent thermal denaturation, which was caused by the unfolding of the protein molecules at high temperatures, often driven by an increase in the configurational entropy of the polypeptide chain. The T_d_ value of the PPI progressively decreased to 107.9 °C when the EGCG concentration was up to 100 mM (Figure 6). This result indicates that EGCG interacted with the PPI and reduced its thermal stability. This may be due to the binding of EGCG to PPI being shown to reduce the α-helix structure of the PPI (Section 3.1.4), leading to the disruption of hydrophobic and electrostatic interactions [40,41]. Our previous study also reported that the T_d_ value of zein-EGCG complexes was slightly lower than that of protein [42].

### 3.3. Molecular Docking Analysis

**Figure 7:**
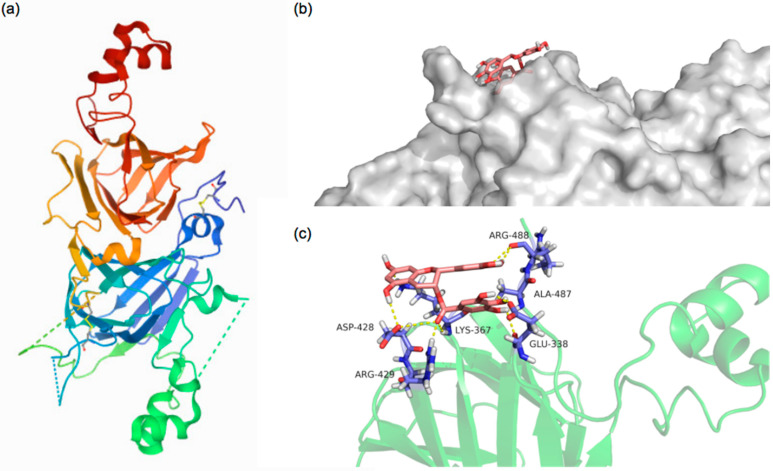
The 3D structure of Entity 1 in the PPI (**a**). The best docked surface structure (**b**) and the best docked conformation (**c**) for the complex of PPI with EGCG. The pink stick structure expresses the catechins, the purple stick represents the amino acids residues, and the yellow dotted line represents hydrogen-bonding.

Molecular docking is an effective method of investigating protein–ligand interactions [32]. In this study, the amino acid residues, numbers of hydrogen bonds, and interaction distances were obtained for the PPI-EGCG interaction by selecting the protein–polyphenol conformation with the lowest energy score, since this is the most likely interaction mode. Molecular docking analysis calculated that the binding energy of PPI with EGCG was −4 kcal/mol, which indicated that the protein and polyphenol could spontaneously interact, providing a molecular basis for the binding of polyphenols to PPI [43]. Figure 7a shows the 3D structure of Entity 1 in the pea protein. Figure 7b shows the preliminary protein surface structure and the docking state of PPI and EGCG. It suggests that polyphenol molecules may interact with specific regions on protein surfaces. The docking results show that EGCG interacted with the amino acid residues GLU-338, LYS-367, ASP-428, ARG-429, AlA-478, and ARG-448 of PPI, resulting in twelve hydrogen bonds with lengths varying from 1.7 to 3.5 Å (Figure 7c). These results therefore support the hypothesis that the hydroxyl groups in the EGCG structure can form hydrogen bonds with the surface residues of the pea protein, which corresponds to the experimental results shown earlier.

### 3.4. Physicochemical Property Analysis of PPI-EGCG Complexes

#### 3.4.1. Surface Hydrophobicity (H_0_)

**Figure 8:**
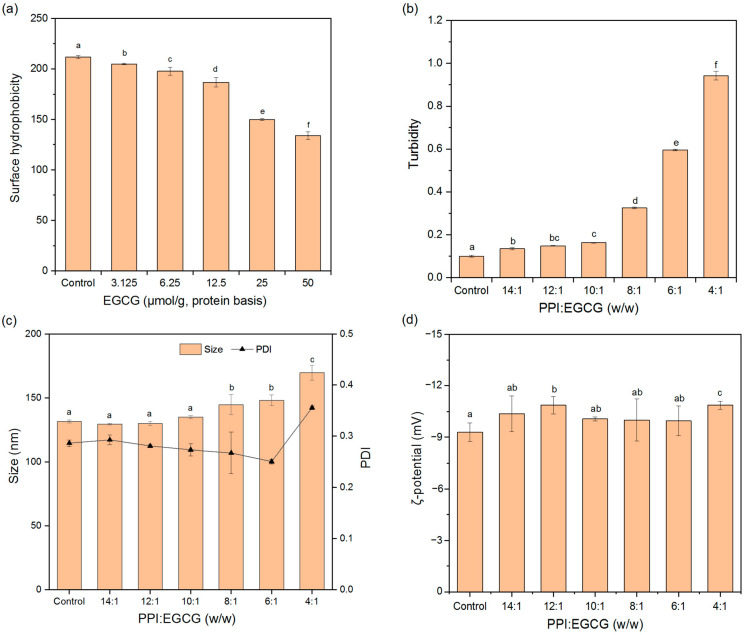
Surface hydrophobicity (**a**), turbidity (**b**), particle size (**c**) and zeta-potential (**d**) of PPI and PPI-EGCG complexes. The different letters represent the fact that the difference is significant (*p* < 0.05).

Surface hydrophobicity can reflect the non-polar hydrophobic groups on the surface of protein molecules, and it can also impact the physical, chemical, and functional characteristics of proteins, such as their solubility, emulsifying, and gelling properties [41]. ANS can bind to hydrophobic groups, which changes its fluorescence intensity. For this reason, it has widely been used to detect the surface exposure of hydrophobic sites and to study configurational changes in proteins. As shown in Figure 8a, EGCG addition reduced the H_0_ of PPI, suggesting that the polyphenols either bound to hydrophobic groups on the surfaces of PPI, or induced changes in the conformation or aggregation state of the proteins and reduced their surface hydrophobicity [44]. EGCG has both hydrophobic and hydrophilic parts, so it may be able to bind to non-polar regions on proteins and increase their hydrophilicity [45]. This increase in hydrophilicity may be beneficial for some applications of pea proteins.

#### 3.4.2. Turbidity

Turbidity measurements can deliver useful knowledge about protein aggregation since the degree of light scattering depends on particle size and concentration. The turbidity of the composite system increased with increasing EGCG content. Initially, the turbidity increased gradually as the mass ratio of PPI-to-EGCG was increased up to 10:1, but then, it increased more steeply when the EGCG concentration was increased further (Figure 8b). This effect suggests that the polyphenols could promote protein aggregation at high concentrations, leading to the formation of relatively large EGCG-PPI complex particles. It would be fruitful to examine the nature of these particles in future studies.

#### 3.4.3. Particle Properties

The particle diameters of all the systems were between 100 and 200 nm, and there was no significant change until the PPI-to-EGCG mass ratio reached 10:1, which agreed with the turbidity data (Figure 8c). The polydispersity index (PDI) values of all the systems were below 0.3 except for the PPI-EGCG complexes with the highest polyphenol concentrations (4:1 *w*/*w*), suggesting that the composite particles formed were relatively uniform in their dimensions. In addition, the addition of EGCG had little effect on the zeta-potential of the complex particles (Figure 8d). This effect is probably because the EGCG molecules were uncharged and the dominant interactions between EGCG and PPI were not electrostatic in origin. As a result, the surface charge of PPI did not change noticeably after binding the polyphenols.

### 3.5. Interfacial Property Analysis of PPI-EGCG Complexes

#### 3.5.1. Foaming Properties

A number of foods consist of gas bubbles dispersed in an aqueous medium, such as whipped cream, beer foams, and some desserts. However, the gas bubbles in food foams often breakdown due to coalescence and Ostwald ripening. Some proteins can rapidly adsorb to the surfaces of gas bubbles, where they unfold and crosslink at the air–water interface, resulting in the formation of a viscoelastic coating that improves foam formation and stability [29].

**Figure 9:**
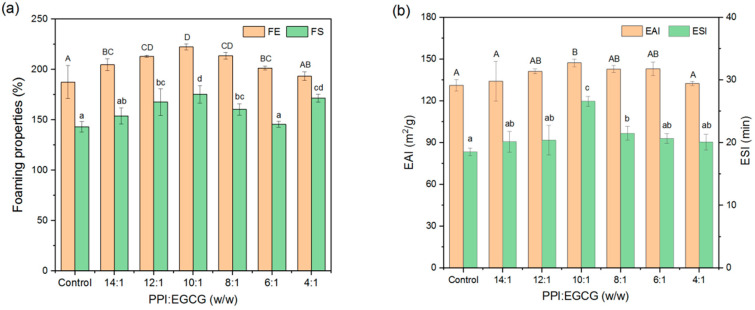
Foaming (**a**) and emulsifying (**b**) properties of PPI and PPI-EGCG complexes. The different letters represent that the difference is significant (*p* < 0.05).

Foam expansion (FE) and foam stability (FS) depend on the film-forming properties of proteins at gas–liquid interfaces. With the addition of EGCG, the FE and FS of systems first increased, and then, decreased (Figure 9a). The best foaming properties were observed for the complexes consisting of a PPI-to-EGCG mass ratio of 10:1. This phenomenon may be due to the complexes produced at optimum polyphenol concentrations having better water solubility characteristics and diffusing to the gas–liquid interface more rapidly. However, when the polyphenol concentration was too high, the proteins aggregated, which reduced their ability to adsorb to the gas–liquid interface and stabilize foams [19]. The data obtained from the foam height measurements suggest that the foam stability was highest at a PPI/EGCG mass ratio of 4:1. However, rapid protein precipitation and bubble collapse were observed in this system after shearing was stopped, indicating that it was extremely unstable (Figure S4).

#### 3.5.2. Emulsifying Properties

Proteins have not only hydrophilic groups but also lipophilic groups, which can be adsorbed on the surface of oil droplets to reduce interfacial tension and provide protection; so, they are suitable as emulsifiers to stabilize emulsions [46]. The emulsifying properties can be used to evaluate the ability of the proteins and protein–polyphenol complexes as emulsifiers. Similarly, the EAI and ESI values of the protein first increased, and then, decreased, and the maximum value was observed at a PPI-to-EGCG mass ratio of 10:1 (Figure 9b). This trend has also been reported in the research of Cui et al. [44] and Jia et al. [18]. These results indicate that adding intermediate concentrations of polyphenols can improve emulsifying properties. However, adding too much can produce protein aggregation; thus, their ability to adsorb to the oil–water interface is reduced. The improvement in the emulsifying properties observed at intermediate polyphenol concentrations may be because the solubility, adsorption, and repulsive forces of protein increased in the presence of the polyphenols [47].

## 4. Conclusions

This study has shown that PPI and EGCG are able to form molecular complexes, which is attributed to the strong physical attractive interactions between the protein and the polyphenol. The interaction between PPI and EGCG was a spontaneous and exothermic process, which appeared to be driven by hydrogen bonds as well as van der Waals forces. The addition of EGCG induced partial unfolding of the pea proteins, causing their secondary structure to become more disordered and reducing their surface hydrophobicity. An optimized (intermediate) EGCG level was shown to improve the foaming and emulsifying properties of the protein. However, if the EGCG level was too high, the proteins aggregated, which reduced their functionality. Our results showed that EGCG addition could improve the physicochemical properties and functional performance of pea proteins, which may broaden their utilization in foods and beverages. Because EGCG has both antioxidant and nutraceutical activities, the formed EGCG–pea protein complexes may be useful as antioxidant or nutraceutical emulsifiers.

## Figures and Tables

**Table 1:** The Stern–Volmer quenching constants of EGCG compounded to PPI.

T(K)	K_sv280nm_(L·mol^−1^)	R^2^	K_q280nm_(L·mol^−1^s^−1^)	K_sv295nm_(L·mol^−1^)	R^2^	K_q295nm_(L·mol^−1^s^−1^)
298	2.796 × 10^4^	0.991	2.796 × 10^12^	2.874 × 10^4^	0.988	2.874 × 10^12^
304	3.312 × 10^4^	0.997	3.312 × 10^12^	3.049 × 10^4^	0.998	3.049 × 10^12^
310	3.314 × 10^4^	0.995	3.314 × 10^12^	3.564 × 10^4^	0.997	3.564 × 10^12^

**Table 2:** The binding constant (K_a_) and number of binding sites (n) of EGCG bound to PPI.

T(K)	K_a280nm_(L·mol^−1^)	n_280nm_	R^2^	K_a295nm_(L·mol^−1^)	n_290nm_	R^2^
298	1.09 × 10^5^	1.14	0.999	2.52 × 10^5^	1.00	0.995
304	4.80 × 10^5^	1.04	0.971	3.86 × 10^5^	1.03	0.997
310	5.00 × 10^5^	1.05	0.987	6.37 × 10^5^	1.07	0.973

**Table 3:** Secondary structure fractions of PPI with different EGCG concentrations.

EGCG (10^−5^ mol/L)	α-Helix (%)	β-Sheet (%)	β-Turn (%)	Random Coil (%)
0.00	27.0 ± 0.5 ^a^	16.2 ± 0.4 ^a^	21.9 ± 0.1 ^a^	35.0 ± 0.1 ^a^
2.50	26.9 ± 0.7 ^a^	16.8 ± 0.4 ^ab^	21.7 ± 0.2 ^ab^	34.6 ± 0.2 ^b^
5.00	26.6 ± 0.6 ^a^	17.1 ± 0.3 ^b^	21.7 ± 0.2 ^ab^	34.6 ± 0.2 ^b^
7.50	25.1 ± 0.3 ^b^	19.3 ± 0.1 ^c^	21.5 ± 0.1 ^c^	34.1 ± 0.1 ^c^
10.0	24.7 ± 0.7 ^b^	19.7 ± 0.4 ^c^	21.6 ± 0.2 ^ab^	34.0 ± 0.1 ^c^

Note: Different letters in the same column represent that the difference is significant (*p* < 0.05).

## Data Availability

Data is contained within the article and supplementary material.

## Supplementary Materials

The following supporting information can be downloaded at: https://www.mdpi.com/article/10.3390/foods11182895/s1, Figure S1: The Stern–Volmer curves of PPI at 280 nm (a) and 295 nm (b) with different EGCG concentrations; Figure S2: The double logarithmic curves of PPI at 280 nm (a) and 295 nm (b) with different EGCG concentrations; Figure S3: Deconvolution of the spectra of PPI (a) and PPI-EGCG complexes with 25 µM (b), 50 µM (c) EGCG. The solid line represents the spectrum measured with λexc. = 280 nm. The dotted line represents the spectrum of the same sample measured with λexc. = 295 nm, while the dashed line is the same spectrum normalized to match the intensity of the spectrum to be deconvoluted at 400 nm. The dash-dot line reports the Tyr contribution, obtained via difference; Figure S4: The foam morphology at 0 min (a) and after 30 min (b) of PPI and PPI-EGCG complexes.
