# Supplementary material for: Structural Characterization and Evaluation of Interfacial Properties of Pea Protein Isolate–EGCG Molecular Complexes
Source: Foods. 2022 Sep 18;11(18):2895. doi: 10.3390/foods11182895 (PMC9498586; doi:10.3390/foods11182895)
Supplement: Supplementary file 1 [file foods-11-02895-s001.zip › foods-1821796-supplementary.pdf]

Supplementary Materials

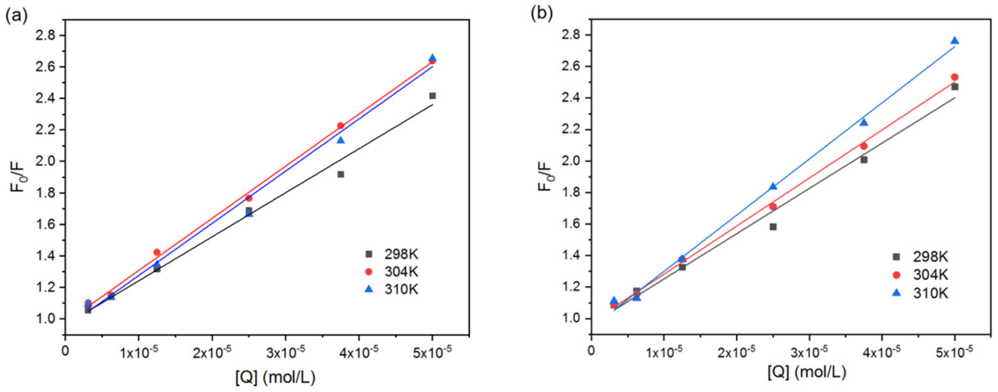

Figure S1: The Stern-Volmer curves of PPI at 280 nm (a) and 295 nm (b) with different EGCG concentrations.

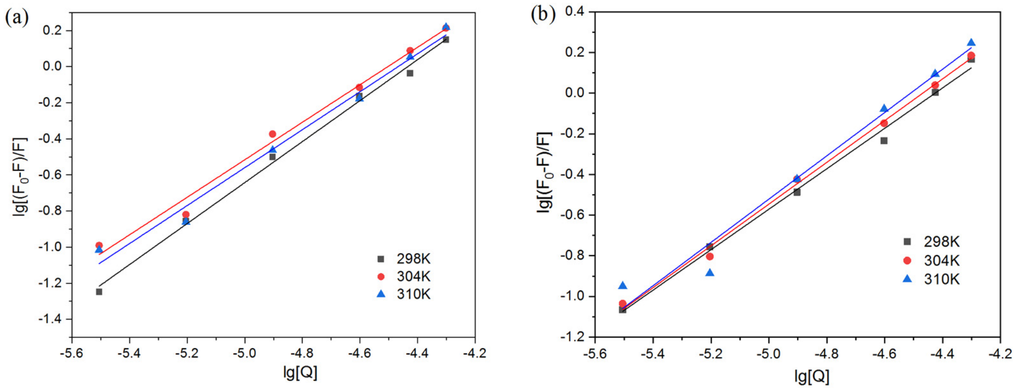

Figure S2: The double logarithmic curves of PPI at 280 nm (a) and 295 nm (b) with different EGCG concentrations.

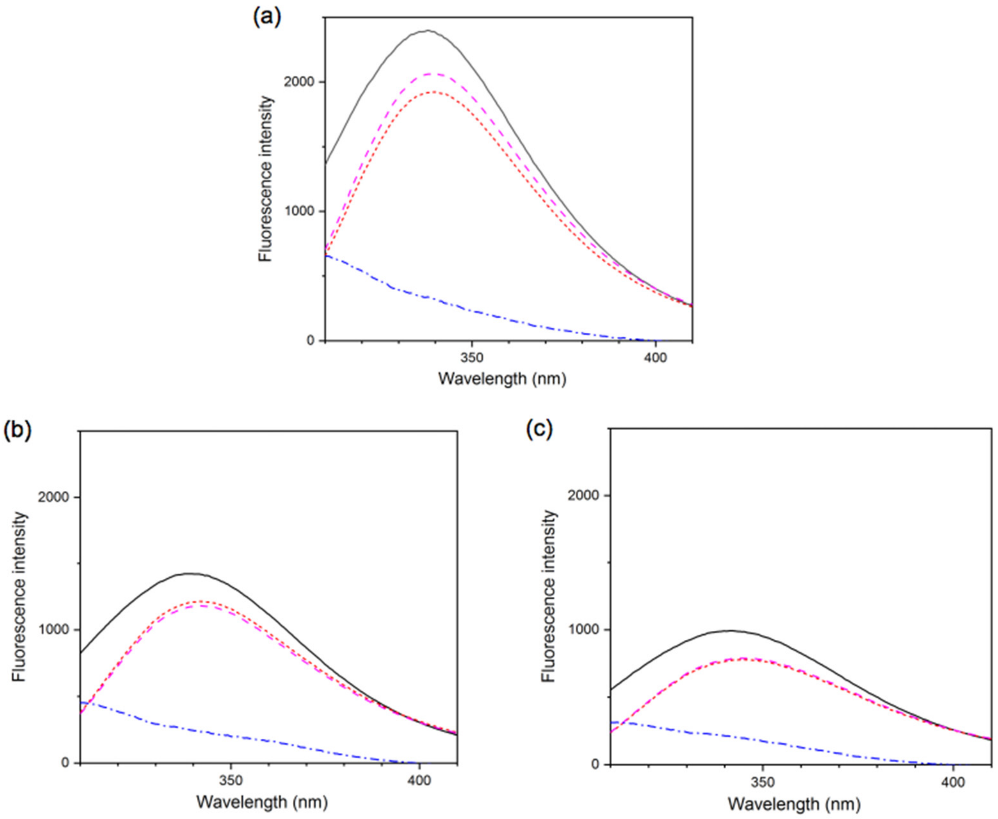

Figure S3: Deconvolution of the spectra of PPI (a) and PPI-EGCG complexes with 25 μM (b), 50 μM (c) EGCG. The solid line represents the spectrum measured with λexc. = 280 nm. The dotted line represents the spectrum of the same sample measured with λexc. = 295 nm, while the dashed line is the same spectrum normalized to match the intensity of the spectrum to be deconvoluted at 400 nm. The dash-dot line reports the Tyr contribution, obtained by difference.

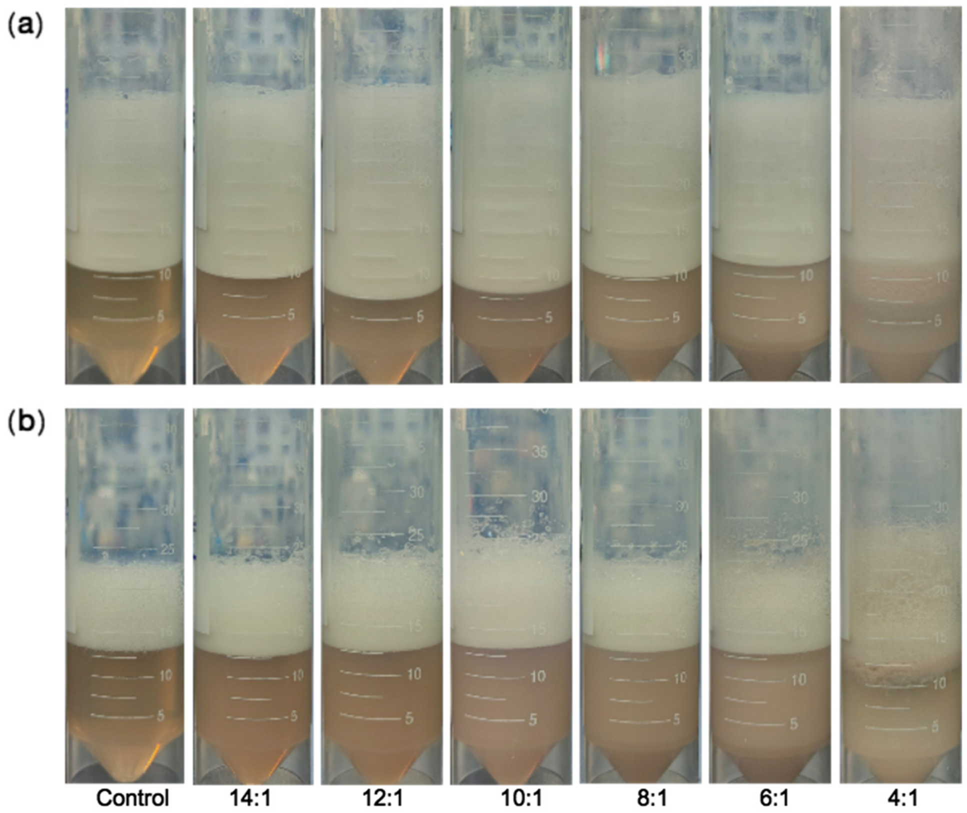

Figure S4: The foam morphology at 0 min (a) and after 30 min (b) of PPI and PPI-EGCG complexes.
